# Activated Protein Kinase C (PKC) Is Persistently Trafficked with Epidermal Growth Factor (EGF) Receptor

**DOI:** 10.3390/biom10091288

**Published:** 2020-09-07

**Authors:** Carol A. Heckman, Tania Biswas, Douglas M. Dimick, Marilyn L. Cayer

**Affiliations:** 1 Department of Biological Sciences, 217 Life Science Building, Bowling Green State University, Bowling Green, OH 43403, USA; tbiswas@bgsu.edu; 2 Department of Physics & Astronomy, 104 Overman Hall, Bowling Green State University, Bowling Green, OH 43403, USA; ddimick@bgsu.edu; 3 Center for Microscopy & Microanalysis, 217 Life Science Building, Bowling Green State University, Bowling Green, OH 43403, USA; mcayer@bgsu.edu

**Keywords:** receptor tyrosine kinase, slow endocytic recycling, annexin, cortactin, vesicle trafficking

## Abstract

Protein kinase Cs (PKCs) are activated by lipids in the plasma membrane and bind to a scaffold assembled on the epidermal growth factor (EGF) receptor (EGFR). Understanding how this complex is routed is important, because this determines whether EGFR is degraded, terminating signaling. Here, cells were preincubated in EGF-tagged gold nanoparticles, then allowed to internalize them in the presence or absence of a phorbol ester PKC activator. PKC colocalized with EGF-tagged nanoparticles within 5 min and migrated with EGFR-bearing vesicles into the cell. Two conformations of PKC-epsilon were distinguished by different primary antibodies. One, thought to be enzymatically active, was on endosomes and displayed a binding site for antibody RR (R&D). The other, recognized by Genetex green (GG), was soluble, on actin-rich structures, and loosely bound to vesicles. During a 15-min chase, EGF-tagged nanoparticles entered large, perinuclear structures. In phorbol ester-treated cells, vesicles bearing EGF-tagged nanoparticles tended to enter this endocytic recycling compartment (ERC) without the GG form. The correlation coefficient between the GG (inactive) and RR conformations on vesicles was also lower. Thus, active PKC has a Charon-like function, ferrying vesicles to the ERC, and inactivation counteracts this function. The advantage conferred on cells by aggregating vesicles in the ERC is unclear.

## 1. Introduction

### 1.1. Signaling from the EGFR

Epidermal growth factor (EGF) receptor (EGFR) is a member of a family of 170–185 KDa transmembrane receptors, also known as the human EGF receptor (HER) or avian erythroblastosis oncogene B (ErbB) family. Signaling from these receptors drives cell growth, so their mutation and overexpression promote tumor formation and serve as prognostic indicators of cancer progression. Although EGFR is the target of many anti-cancer drugs, resistance to these drugs inevitably develops (review [1]). Drug resistance is often acquired through a mutation in the receptor itself or through changes that alter receptor processing so as to enhance signaling from the receptor (review [2]). As more than one member of this receptor family can coexist in a cell, together they assemble a scaffold on which a vast variety of other molecules can be assembled. Thus, the initial signaling complex comprises a large number of possible states [3]. After activation by ligand ligation, a receptor can be directed into a variety of different compartments, and signaling can continue from some of these compartments. If signaling is enabled in an internal compartment, internalization will prolong signaling from the EGFR until it is finally redirected to the lysosome. If prohibited, signaling will cease until the receptor is recycled into another permissive compartment. Whereas many of the mechanisms that govern processing are known, the triggers that redirect receptor trafficking from one path to another are unknown. As workers have postulated that one of these triggers is protein kinase C (PKC) (see Section 1.2*. Does PKC regulate EGFR traffic?*), we traced the co-internalization of EGFR and PKC.

### 1.2. Does PKC Regulate EGFR Traffic?

Although PKC is rarely if ever directly attached to the receptors of the EGFR family (review [3]), it alters the EGFR trafficking pattern. The mechanism for this may lie in its ability to phosphorylate the Thr654 residue of the receptor [4]. How the traffic pattern is affected, however, depends on the temporality of PKC intervention. If phosphorylation occurs rapidly after receptor ligation [5,6], it causes agonist-dependent desensitization. This may occur by reducing the receptor’s affinity for ligand, its dimerization, and/or its autophosphorylation on tyrosine, thereby preventing its assembly into higher-order aggregates and inhibiting internalization and recycling [7]. In other cases, phosphorylation takes place concurrently or after internalization [8,9,10]. In this case, treatment with phorbol ester tumor promoters, which are surrogates of diacylglycerol (DAG), causes the retention of receptors inside the cell, although their degradation is delayed [11,12].

**Figure 1:**
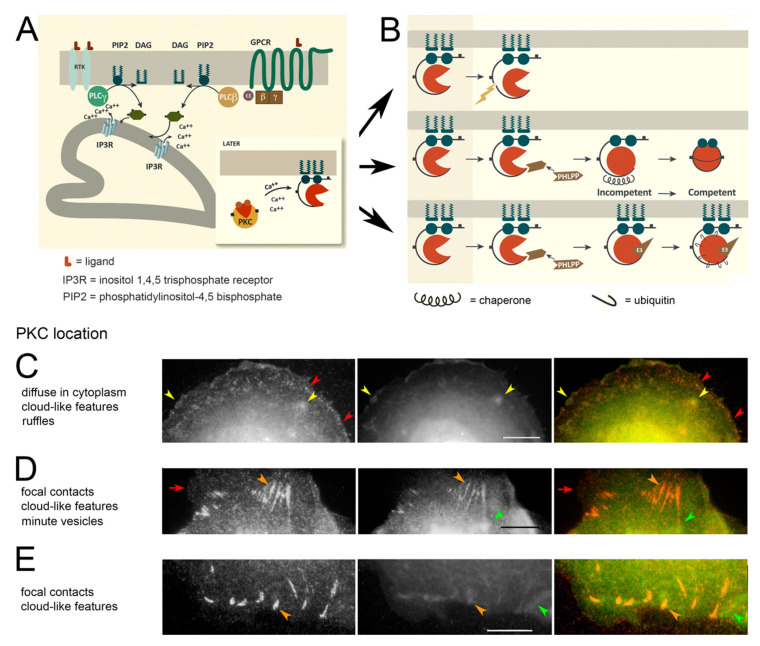
Protein kinase C (PKC) activation and trafficking. (**A**) PKC activation is initiated by receptor tyrosine kinases (RTKs) (left) or G protein-coupled receptors (GPCR) binding their respective ligands (L). This activates a phospholipase C (PLCβ, PLCγ) and causes the hydrolysis of L-α-phosphatidylinositol(4,5)bisphosphate (PIP2), yielding DAG along with inositol (1,4,5)trisphosphate which activates its receptor (IP3R) in the endoplasmic reticulum. IP3R allows the release of Ca^++^ into the cytoplasm. Inset: The combination of DAG and Ca^++^, or just DAG for the novel isoforms, causes the protein to unfold revealing phosphatidylserine-binding sites (green circles). This allows PKC to dock on a membrane and access its substrates. (**B**) Possible fates of PKC after activation. The protein must be membrane-bound to maintain enzymatic activity. After PKC hydrolysis, the enzymatically active half becomes soluble (top panel) but has a short half-life. The hydrophobic motif (see Figure S1A,B) is dephosphorylated by Pleckstrin Homology Domain Leucine-rich repeat Protein Phosphatase (PHLPP), inactivating the enzyme. This form can be removed from the membrane by a chaperone (middle panel). PKC can be degraded in an inactive form by ubiquitination by an E3 ligase (bottom panel). (**C**–**E**) Vinculin (left panel, red) colocalized with PKC (middle panel, green), in untreated cells (see Section 2.3*. Localization by Indirect Immunofluorescence*). (**C**) PKC is on actin-rich ruffles and cloud-like features (yellow arrowheads) but absent from some focal contacts (red arrowheads). (**D**) PKC binds to some cloud-like features without vinculin (green arrowheads). It is colocalized with vinculin in most focal adhesions (orange arrowheads) but is absent occasionally (red arrowheads). (**E**) PKC binds to focal contacts (orange arrowheads) but is also found between these structures in a form that is free of vinculin (green arrowheads). (**C**–**E**) Bars = 10 µm.

PKC is a family of serine-threonine kinases, comprising some 11 isoforms which are activated downstream of hormone and growth factor receptors. EGFRs belong to the class of receptor tyrosine kinases (RTKs). They activate a phospholipase C, PLCγ, which causes hydrolysis of L-α-phosphatidylinositol(4,5)bisphosphate (Figure 1A) in the plasma membrane. This in turn causes the release of second messengers, DAG and calcium, to activate the conventional class (α, β, and γ) of PKCs. PKCs of the novel class, (δ, ε, η and θ) are activated by DAG, cis-unsaturated fatty acids, phosphatidic acid, or L-α-phosphatidylinositol-3,4,5-trisphosphate. These mechanisms, along with a parallel pathway operating downstream of G protein-coupled receptors, represent the canonical pathways of PKC activation (Figure 1A). PKC docks on membranes through the combined effect of DAG, phosphatidylserine binding sites, and (for the calcium-activated isoforms) calcium [13,14].

It is well known that fluid-phase endocytosis is enhanced by PKC activation, and that it causes an expansion of the volume of the endosomal compartments [15]. One of the compartments affected is a storage and recycling compartment called the endocytic recycling compartment (ERC) or pericentrion (review [16]). Accumulation of integral membrane proteins in the ERC is purportedly regulated by PKC isoforms of both the conventional and novel classes [9,12,17,18]. The mutation of PKC-γ, which causes the neurodegenerative disease, spinocerebellar ataxia type 14, involves a failure of PKC to redirect vesicles into the ERC [18], which argues for a physiological significance of this compartment. However, there is reason to question that PKC has any specific role in regulating endosome trafficking. The enzymatic activity of PKC is subject to tight regulatory controls, but it has not been widely acknowledged as a regulator of vesicle traffic. For example, it is scarcely mentioned in recent publications [19] and reviews [16,20,21] on endocytic trafficking. If excessive signaling downstream of receptors drives a process of vesicle “pile-up”, PKC may set it in motion without having any further role in its regulation. Since the PKCs are bound to the plasma membrane in their activated form, it is possible that they phosphorylate the EGFR through a hit-and-run mechanism and do not remain on endocytic vesicles bearing EGFR. On the other hand, if PKC molecules only joined the vesicles upon their internalization, they would not be colocalized with the EGFR at the cell surface.

Here, we test these possibilities by determining whether PKC is associated with receptors early after their ligation with EGF and whether this association persists during subsequent trafficking. To this end, we colocalize the EGFR with PKC-ε using a model system in which PKC-ε activation has already been characterized. It was clear from previous work that, whereas activation occurred by 30 min following treatment of the respiratory airway cells with a phorbol ester, there was little degradation until 5–10 h after treatment. As degradation products could not be identified in immunoprecipitation experiments, they had a short half-life [22]. This enabled us to find intact PKC, which could be in its competent, soluble form or in its enzymatically active form, which is on membranes. We found a persistent relationship of PKC with receptor-bearing vesicles, suggesting that PKC joined the EGFR at the plasma membrane and accompanied it during its travels. However, we found unexpected complexity in the species of PKC recruited to vesicles having EGF-tagged gold particles, which matched phases of the cyclical inactivation and recovery of PKC molecules.

Although this result showed that PKC accompanies the endosomes to the ERC, it failed to reveal the regulatory mechanism. If there is merely a bottleneck in the machinery needed to process the endosomes into the degradation or recycling pathways, the endosomal membrane compartments would pile up and resemble the ERC. Consistent with this interpretation, the markers for the ERC overlap with those of the other endosomal compartments, i.e., early endosomes, sorting endosomes, and late endosomes [23]. PKC is the most specific marker for the ERC (review [24]). Of the other proteins present, Rab11 is considered a marker but is also present in early endosomes and multivesicular bodies (review [21,25,26]). The early endosomal markers, Rab5 and EEA1, and late endosomal markers, Rab7 and Lamp1, accumulate in the ERC [27]. The significance of our findings is that one conformation of PKC, thought to include the enzymatically active molecules, is processed to another during endosomal trafficking. The processed molecules are more loosely bound to vesicles, and the frequency of their attachment to vesicles is much reduced after treatment with phorbol esters. Thus, the development of the ERC is linked to the activation of PKC on vesicles being directed to that destination. This process may be important in signaling and growth control (see Discussion).

## 2. Materials and Methods

### 2.1. Cell Culture and Nanoparticle Exposure

The 1000 W cell line was generated from rat respiratory tract epithelium with 7,12- dimethylbenz(a)anthacene [28] and cultured in Waymouth’s medium containing penicillin, streptomycin, 10% fetal bovine serum (Atlanta Biologicals, Atlanta, Georgia), 0.1 µg/mL insulin and 0.1 µg/mL hydrocortisone (Wihc+10%). For experiments, cells were transferred at a density of 4 × 10^5^ per 60 mm dish and incubated at 37 °C with 5% CO_2_ in air, for 1–2 days. Cells to be labeled with EGF-coupled, Cy3-labeled nanoparticles were cultured on germanium-coated coverslips or Tolansky substrates [29,30]. The samples were placed in serum-free medium at 37 °C for 45 min to maximize receptor display and then transferred to a 1:4 mixture of nanoparticles in serum-free medium at 4 °C for 15–45 min. To follow the course of nanoparticle uptake, we placed some samples back into the original dishes containing Wihc+10% at 37 °C for chase periods of 5–30 min before fixation. To compare nanoparticle processing with and without phorbol ester treatment, the serum-deprived cells were transferred into the nanoparticle mixture at 4 °C for 20–45 min and then transferred back into the original dishes for 15 min. For cultures treated with phorbol 12-myristate 13-acetate (PMA), the agent was made up in ethanol and used at a final concentration of 2 nM. The solvent vehicle alone was added to the control samples. PMA was purchased from LC Laboratories (Woburn, MA, USA). Culture supplies were purchased from ThermoFisher (Carlsbad, CA, USA) unless otherwise noted. Where the cultures were treated with PMA and A23187, the final concentration of A23187 was 1 × 10^−7^ M.

The samples were fixed at 37 °C with 3% formaldehyde, made fresh from paraformaldehyde, rinsed in phosphate-buffered saline (PBS), and stored in PBS at 4 °C as described previously [30]. Controls on binding or uptake of nanoparticles employed nanoparticles before conjugation with EGF.

### 2.2. Preparation of Nanogold

Quantum dots of 1–2 nm size [31], derivatized with bovine serum albumin, were used to determine a favorable chemistry for coupling nanoparticles to EGF. To detect coupling, proteins from fetal bovine serum were adsorbed on a Tolansky substrate, rinsed, and exposed to glutaraldehyde. After rinsing with PBS and deionized water, the solution was replaced with quantum dots which bound to the free aldehyde moieties. The emission spectrum, excited by a 445 nm laser and read in the range 545–1100 nm, was evidence of effective chemical coupling between the nanoparticle and the proteins on the substrate (data not shown).

50-nm colloidal gold nanoparticles, conjugated with Cy3 (cyanine3)-tagged goat anti-mouse immunoglobulin G, were purchased from NanoPartz (Loveland, CO, USA). To couple nanoparticles to EGF, 150 µL of gold suspension (3 × 10^11^ particles) was made up to 1.5 × 10^-7^ M glutaraldehyde in PBS to achieve a molecular ratio of 10,000:1 glutaraldehyde to nanoparticle. The mixture was dialyzed against PBS in a Pierce Slide-A-Lyzer dialysis cassette with 3500 MW cutoff (ThermoFisher, Radnor, PA, USA) for 1–3 h to eliminate free glutaraldehyde. Finally, 2 µg of recombinant mouse EGF (Life Technologies, Carlsbad, CA, USA) was added to the suspension. After 1 h at room temperature, the nanoparticles were spun down at 9000 rpm for 3 min in an Eppendorf 5415 C microfuge. They were rinsed with PBS and recovered by the same procedure. The gold suspensions were stored at 4 °C until use in experiments.

### 2.3. Localization by Indirect Immunofluorescence

To colocalize EGFR and PKC-ε cells were permeabilized with 50 µg/mL digitonin (LC Laboratories, Woburn, MA, USA) made up in 0.1% Triton X-100 in PBS. In later experiments, the digitonin concentration was elevated to 100 µg/mL to reduce the background from soluble PKC.

Samples were exposed to the primary and secondary antibodies as previously described [22] and mounted in 2.5% DABCO made up in 2,2′-thiodiethanol (Sigma-Aldrich, St. Louis, MO, USA). For PKC colocalizations, cells were stained with a rabbit polyclonal antibody against PKC-ε (Genetex, Irvine, CA, USA) followed by a secondary goat fluorescein isothiocyanate-labeled anti-rabbit antibody at a dilution of 1:3000. The Genetex antibody, called Genetex green (GG), was directed against a region within amino acids 358 and 737 (Uniprot ID: Q02156). This was colocalized with the nanoparticles or with a mouse monoclonal antibody against vinculin (hVIN-1, Sigma-Aldrich, St. Louis, MO, USA). To localize the latter, we used a secondary donkey anti-mouse tetra-rhodamine (TRITC)-labeled antibody (Jackson ImmunoResearch). When dual PKC localizations were conducted, a primary sheep anti-PKC-ε (R&D Systems, Minneapolis, MN, USA) was used. We localized this antibody by a procedure called RR, using a secondary Cy3-conjugated donkey anti-sheep IgG (Jackson ImmunoResearch, West Grove, PA, USA). The sheep antibody was directed against a shorter fragment, 580-737. As both anti-PKC antibodies were affinity-purified on columns derivatized with the respective recombinant fragments, the difference in localization was attributed to different epitopes. Figure S1 shows the sequence of PKC-ε along with the topography of the active site with phosphorylation sites at Thr566, corresponding to Thr500 (PKC-βII) and at hydrophobic motif Ser729, corresponding to Ser660 (review [32]). Phosphorylation at these sites “primes” the enzyme and makes it competent to convert substrates. A map of the domains, shown in Figure S1C,D, indicates the most likely conformation of competent but inactive enzyme. This is thought to be the form identified by antibody GG (see below). A map of the epitopes is shown in Figure S1E.

Each PKC-ε molecule is thought to occupy one of three states in resting cells: (1) competent, i.e., “primed” and ready for activation, (2) enzymatically active, or (3) in the process of being recycled. While enzymatically active, the protein must be associated with a membrane. As a result that PKC-ε is degraded if cells are exposed to phorbol ester for over 5 h [22], only about one-half of the original PKC-ε remains after 10 h. Localizations with GG show that this is mainly soluble (Figure S1F). Thus, the GG antibody recognized at least one soluble form of PKC-ε.

### 2.4. Image Acquisition and Analysis Procedures

The samples were viewed with a 63× or 100× lens in a Zeiss Axiophot and images acquired with Roper Princeton Instruments RTE/CCD camera and IBM-PC running MetaMorph 4.6r5 software. For confocal imaging, a Leica DMI3000B inverted microscope (Leica Microsystems, Buffalo Grove, IL, USA) equipped with a Lumen Dynamics X-Cite light engine and Spectra X LED source (Lumencor, Beaverton, OR, USA), X-Light spinning-disk confocal unit (CrestOptics, Rome, Italy) and Rolera Thunder cooled CCD camera with back-thinned, back-illuminated, electron-multiplying sensor (QImaging, Surrey, British Columbia, Canada) was used.

The coincidence of nanoparticles with spherical concentrations of PKC was compared by analyzing overlaid images taken from samples that were rewarmed in Wihc+10% with or without PMA for 15 min. The number of structures with coincident labels was divided by the total number of nanoparticles identified in selected areas of each micrograph.

### 2.5. Image Quantification, Correlation Analysis, and Statistics

The size of particles stained with the GG and RR procedures was measured on images from thin, peripheral portions of the cells. The selection of these areas obviated the complication of multiple layers of structures, which would otherwise lead to overlapping layers of particles. Pearson correlation coefficients were obtained for matched images in the green and red channels. Colocalization Finder [33] plugin, running under ImageJ [34], was used. With this method, the background exterior to the cells and parts of the interior could be excluded, and the analysis was made specific to the features of interest.

Microsoft Excel was used to calculate averages and standard deviations. The significance of differences between means was analyzed by the two-tailed Student *t*-test. All error bars represent plus or minus one standard error of the mean.

## 3. Results

### 3.1. PKC Locations Are Dictated in Part by Actin Binding

As a result that PKC-ε has an actin-binding motif at amino acids 223–228, we sought to determine whether actin binding was influencing enzyme location by comparing PKC to a protein whose actin-binding characteristics are well understood. We chose vinculin for this purpose because both the actin-binding domain and a phosphorylation site for PKC are exposed when the vinculin molecule unfolds [35]. The actin-binding domain of vinculin is critical to its functions, for example, slowing retrograde flow of the actin subunits [36]. Using the GG procedure for localizing PKC, we imaged PKC in cells treated with a phorbol ester activator compared to untreated cells. The specific component of staining, i.e., that dependent on primary antibody binding, may represent both the resolubilized and competent forms of PKC in the model (Figure 1A,B). The antibody against vinculin was mainly localized to discrete structures, especially focal contacts (Figure 1C). One location, where PKC was concentrated as well, was in cloud-like formations. These sometimes contained both proteins (Figure 1C, yellow arrowheads) and sometimes mainly or solely PKC (Figure 1D,E, green arrowheads). Finally, the GG conformation was present on small vesicles Figure 1D and Figure S1F.

Although the cloud-like structures resembled ruffles in texture, the clouds could be present far away from the cell edge where ruffles are rarely found (Figure 1C). In cloud-like features, as well as ruffles, PKC was often present with actin and vinculin (Figure S2A,B). Thus, these may represent actin-rich structures binding PKC through its actin-binding domain. Either PKC or vinculin could be absent, as noted above. Although there was a correlation of ~0.5 between PKC and vinculin in images, further analysis did not reveal any effect of the phorbol ester activator (Figure S2C), suggesting that binding of the GG form to these structures was not greatly affected by PKC activation.

After long-term exposure to a phorbol ester activator, some of the PKC-ε was degraded as mentioned above and the remainder was largely soluble (see Section 2.3*. Localization by Indirect Immunofluorescence)*. When the soluble form of PKC predominated over the other forms localized, this could be recognized by its pattern of exclusion from the larger organelles. This pattern gave the cells a mottled or holey appearance (Figure S1F). Controls exposed to irrelevant primary antibodies, followed by the same secondaries as mentioned above, showed no specific staining (Figure S3A,B).

### 3.2. PKC Is Recruited to Membranes with EGF-Tagged Gold Particles

**Figure 2:**
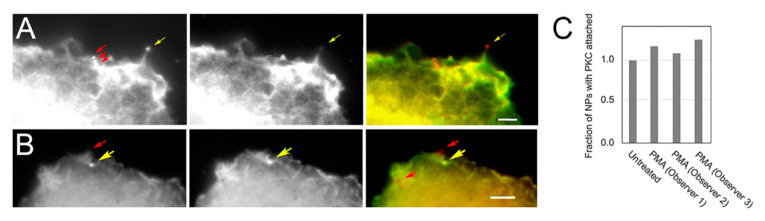
Colocalization of epidermal growth factor (EGF)-tagged nanoparticles and PKC-ε (**A**,**B**) EGF- and Cy3-labeled nanoparticles (left panel, red), PKC (center panel, green), and overlaid images (right panel) of cells fixed 5 min after re-warming. (**A**) Three single nanoparticles (red arrows) are present as well as a cluster of nanoparticles at the tip of a filopodium (yellow arrows). (**B**) PKC is concentrated at a single, in-focus nanoparticle (yellow arrows) while out-of-focus nanoparticles can be detected on ruffles (red arrows). (**C**) Counts of EGF-coated nanoparticles overlaid with PKC localized by the Genetex green (GG) procedure. The phorbol ester exposure had little effect on recruitment of the GG form of PKC to nanoparticles. (A–B) Bars = 3 µm.

The above localizations employed the GG antibody, recognizing the amino acid sequence, 358–737, and the same antibody was used in combination with EGF-tagged nanoparticles to track EGFR. Cells were exposed to EGF-tagged nanoparticles in the cold, which enabled the particles to bind to the receptors while preventing internalization. Then, the preconditioned medium was restored and the cells left in the absence of nanoparticles for varying lengths of time. As shown in Figure 2A,B, nanoparticles were bound at the periphery of the cell on ruffles and on filopodia, as described previously [37,38]. As a result of the limited depth of field of high-magnification lenses, nanoparticles were less sharp in the image where the cell rose steeply from the substrate, for example, on ruffles (Figure 2B). The nanoparticles were mainly 50 nm in diameter, indicating that they were typically bound as single particles (Figure 2A,B). After restoration to warm medium, the cells were maintained in the presence or absence of PMA to trace the nanoparticles in the cytoplasm. To allow us to visualize the particles at high magnification in PMA-treated cells, we administered the PMA with calcium ionophore, A23187, which depressed the ruffling activity. There was little change in the number of nanoparticles that coincided with PKC staining when cells were treated with PMA (Figure 2C). Nanoparticles coated with goat anti-mouse immunoglobulin G were not taken up by the cells (Figure S3C).

### 3.3. Vesicles Recruit PKC Displaying the RR Epitope and Retain It during Internalization

**Figure 3:**
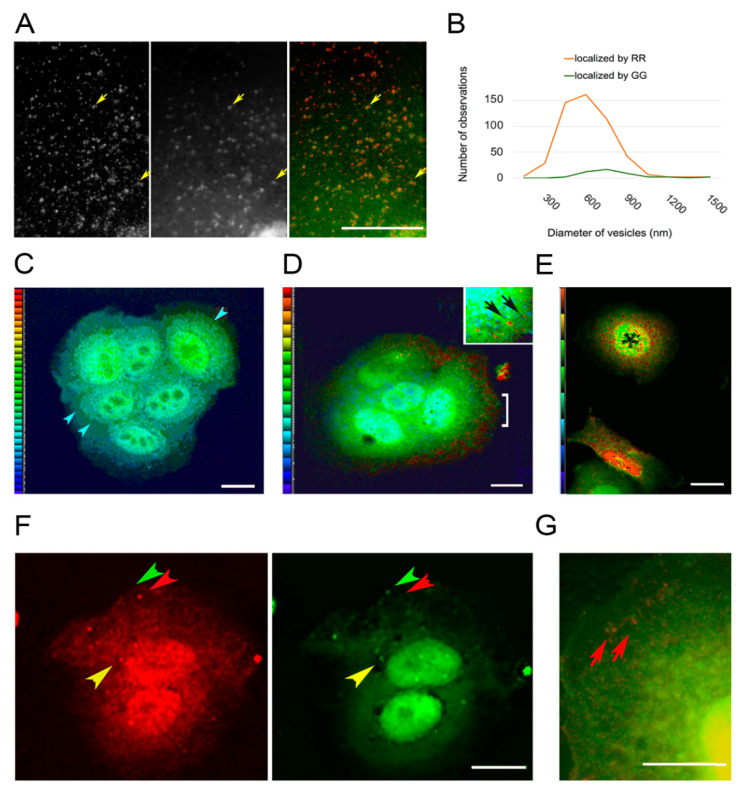
Two anti-PKC antibodies directed against epitopes identified by procedures GG and RR. (**A**) In untreated cells, the RR form (left panel, red) of PKC predominates on vesicles at the extreme cell edge (top), whereas the GG form is rarely present at the cell edge. Vesicles binding both forms are present in the cell interior (yellow arrowheads). (**B**) Size distribution of PKC-bearing vesicles, showing a peak at 400–600 nm for RR vesicles and a broad distribution centered around 750 nm for GG vesicles. (**C**–**E**) Heat maps with a color scale of blue to red indicating increasing GG/RR or RR/GG ratios. (**C**) GG/RR ratio shows a higher concentration in the lamella of untreated cells (blue arrowheads). (**D**) In a cell treated with PMA for 30 min, the RR/GG ratio is highest on vesicles near the cell edge (bracket). Inset: Highest ratio, indicated in red, shows that PKC is at the surface of hollow structures (arrows). (**E**) RR/GG ratio image of untreated cells showing a low ratio in one cell (asterisk) and a high ratio in the cell below. (**F**) Localization of the PKC RR (left, red) and GG (right, green) epitopes in cells after 30 min of PMA treatment. Some vesicles are coated with only the RR form (red arrowheads). Some have only the GG form (green arrowheads), and some have both forms (yellow arrowheads). (**G**) In a cell exposed to PMA for 10 h, PKC localized by the RR antibody (red) is in large vesicles near the cell edge (red arrows) but when localized by the GG antibody (green), diffuse in the cytoplasm. Bars = 10 µm.

These data showed that the EGF-tagged nanoparticles were bound to EGFR and the nanoparticles were also bound on sites recruiting PKC-ε. That the phorbol ester activator did not affect recruitment of PKC (Figure 2C), however, suggested that PKC was not regulating the formation of early endosomes. This was due to the fact that these experiments recognized PKC by the GG procedure, and this form did not seem to be tightly bound to membranes despite PKC’s activation requiring lipids. To better understand the maturation of the vesicles, we localized PKC using two anti-PKC antibodies directed against different epitopes. The RR staining intensity was typically higher than that of GG, especially at the cell edge where very few vesicles exhibited the GG form. Further away from the cell edge, there were vesicles that only displayed the GG form (Figure 3A). This accounted for the apparent lack of effect of PKC activation on the PKC-EGFR colocalization shown in Figure 2C. The difference in size of the coatings was confirmed by analyzing the diameter of the vesicles in the image (Figure 3B). We considered the possibility that the explanation for the difference in compactness of the vesicles’ coatings was that the antibodies differed in affinity for PKC. As the distance from the cell edge increased, however, the distribution of these two forms differed. The sites where they were colocalized (yellow arrowheads, Figure 3A) were more distant from the cell surface, suggesting that the PKC displaying the RR epitope was present on new vesicles, and it was gradually converted to the GG form of the molecule.

The above results suggested that the differing localizations of the GG and RR forms on vesicles were due to physiological factors, rather than a difference in antibody avidity or binding affinity. There were three arguments for proposing that the RR antibody recognized an epitope on the active form of PKC. First, the confinement of antibodies to vesicle edge was thought to reflect the membrane association of the active form, which resembles beads on a string [39]. Secondly, PKC is activated mainly or solely at the plasma membrane, and most vesicles near the cell edge (top of Figure 3A) displayed the epitope identified by RR. Finally, there was little diffuse, cytoplasmic staining with the RR procedure, reflecting the fact that the active form is membrane-bound. The observation that very few vesicles at the cell edge had recruited the more diffuse and loosely associated GG form of coating, suggested that the GG conformation was achieved by processing the RR form. The GG procedure thus recognized a molecule that is transient on vesicles, because it was in the process of being dissociated and recycled.

### 3.4. PKC Conversion from the Form Displaying the RR Epitope to the GG Form of the Molecule

To better understand the GG and RR colocalization, we made ratio images using the maximum projection of intensities from confocal stacks (Figure 3C–E). The GG form of PKC showed an elevated intensity in the cells’ lamella, consistent with its distribution as a soluble component (Figure 3C). The RR form was concentrated around vesicles, especially near the cell edge (Figure 3D). The decreased ratio at locations interior in the cell could be attributed to the shedding or processing of the RR form as vesicles were moving through the cytoplasm (see Section 4.2 *PKC Localization).* We therefore imaged cells after exposure to the PKC activator, PMA. The GG and RR forms were colocalized on some vesicles, as they were detected by both antibodies, but after PMA exposure, the dual localization was rare (Figure 3F, yellow arrowheads). We thought the form recognized by the RR antibody would be greatly reduced in cells treated with PMA for 10 h, because the enzyme undergoes degradation between 5 and 10 h. Dual localization with the GG and RR antibodies showed that, after depletion of the PKC content, very few organelles containing the RR form remained, but the GG form was present in cytoplasm and in perinuclear organelles (Figure 3G).

**Figure 4:**
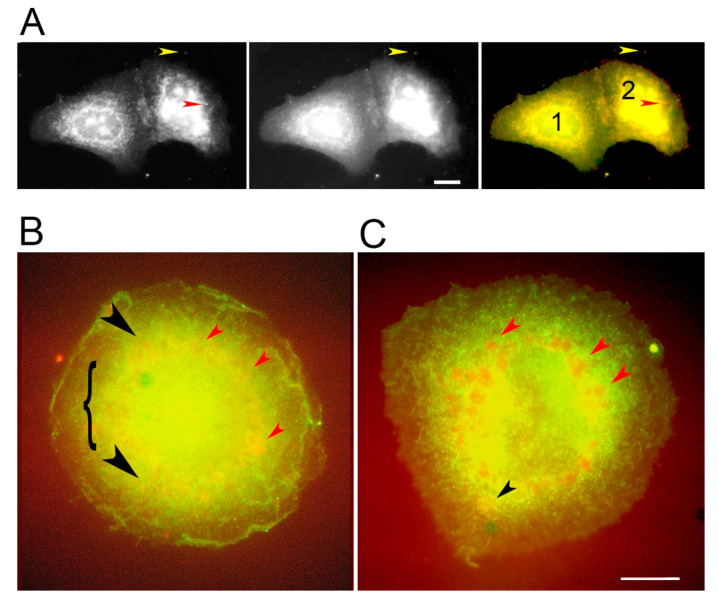
Colocalization of EGF-tagged nanoparticles with the GG form of PKC. (**A**) Projection of confocal planes showing rare locations where there are nanoparticles alone (left panel, red arrowhead) or together with PKC on a filopodium (yellow arrowhead) in PMA-treated cells. PKC (middle) is found with the nanoparticle (yellow arrowhead) and around or above the nucleus. Overlaid images (right) show both labels concentrated in a perinuclear ring (nucleus marked 1) or beside and above the nucleus marked 2. The cytoplasm shows homogeneous staining from the GG form of PKC (green). (**B**) A control cell, untreated during the 15-min chase, shows nanoparticles colocalized with the GG form in perinuclear sites (red arrowheads). Smaller colocalized clusters are found farther from the nucleus (black arrowheads) amidst a concentrated background of soluble PKC (bracket). (**C**) A cell treated with PMA during the 15-min chase shows large clusters of nanoparticles with little PKC GG in perinuclear locations (red arrowheads). A few smaller clusters are found farther away from the nucleus (black arrowhead). The bright green spot is an artifact. Bars = 10 µm.

These results suggested that PKC activation prevents accumulation of the GG form on small vesicles, as would be expected if the molecules displaying the GG epitope were processed from the enzymatically active form. Heat maps showing the RR/GG ratios in untreated cells indicated a wide range of values at the perinuclear sites (Figure 3E), however, so the cell-to-cell variation was large. To further explore the differences caused by PMA exposure, cells were allowed to take up EGF-tagged nanoparticles and then incubated for a chase period of 15 min with or without PMA. The nanoparticles were concentrated in globular or ring-shaped structures around the nucleus and on top of the nucleus, where they were superimposed in images with the GG form of PKC (Figure 4A). The localization of PKC staining intensity to one side of the nucleus, reported by other workers, was only found in 30–45% of treated and untreated cells (see for example cell 2, Figure 4A).

**Figure 5:**
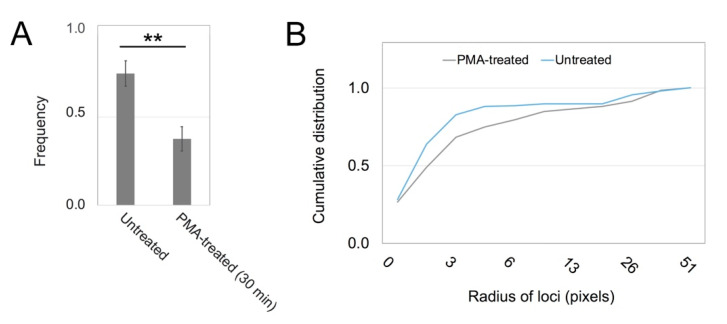
Treatment with PMA inhibits GG-form conversions and enhances nanoparticle clustering in the ECR. (**A**) Representative results from three experiments showing the frequency with which RR-labeled sites coincide with GG labels. (**B**) Cumulative size distribution of Cy3-labeled objects in cells with and without PMA treatment for 15 min. Elevated frequencies in the low size range from the thresholded images indicate that the loci containing gold nanoparticles are slightly smaller. ** means differ by *p* < 0.02.

It appeared that untreated cells had more extensive GG staining in the interior than PMA-treated cells. To study this further, we analyzed the frequency of overlap of RR and GG labels on vesicles. These results showed that the RR forms were more often overlaid by GG-stained loci in untreated cells than in PMA-treated cells (Figure 5A). It was thought that many more molecules were processed into the GG form in untreated cells, causing the display of the GG form to persist on RR-positive vesicles. Conversion of RR to GG occurred less readily after treatment with a PKC activator. Studies were also undertaken to analyze the size of the ECR in images such as shown in Figure 4B,C. Cy3-positive areas, which represented clusters of nanoparticles, were segmented from the images and subjected to particle analysis in ImageJ. The results suggested that loci in this area were more fragmented in the untreated cells than in the PMA-treated cells (Figure 5B).

As mentioned above, the GG form of PKC appeared to be concentrated in the upper half of the cells (Figure 4B). There were large, spherical loci around the nucleus, where the yellow color in overlaid images represented highly concentrated GG form of PKC, colocalized with nanoparticles (Figure 4B). Despite the similar appearance of sites bearing EGF-tagged gold particles in cells treated with the phorbol ester activator of PKC, the colors of the loci in overlaid images from these cells appeared orange rather than yellow (Figure 4C). As a result that this suggested depletion of the GG form, we measured the Pearson correlation coefficient of Cy3 and GG images of perinuclear areas. The results showed that the coefficients were higher in control than in treated cells with a statistical significance of *p* < 0.05. Control samples showed a mean of 0.736 (S.D. 0.074, N = 6) versus treated 0.442 (S.D. 0.273, N = 6) where S.D. represents the standard deviation and N the number of images analyzed.

## 4. Discussion

### 4.1. What Is the ERC?

Receptors and other cell surface proteins are typically directed to the lysosome for degradation or recycled back to the plasma membrane. There are at least two routes of recycling; however, one route entails little delay in being recycled to the cell surface, while the slow route implies the existence of one or more compartments that store vesicles for prolonged periods. The ERC which occupies a perinuclear site near the centrosome is considered one such compartment, but it is not distinctive in terms of morphology and in fact, cannot be identified at all in some cell types. As mentioned above (see Section 1.2*. Does PKC regulate EGFR traffic*?), treating cells with a phorbol ester or with a high concentration of a relevant ligand, such as EGF, causes a hyper-activation of endocytosis, which includes nonreceptor-mediated endocytosis. This may overwhelm the cell’s capacity for sorting vesicles to their destinations, in which case they end up in the perinuclear location. While early endosomal markers are present, indicating vesicles that are in an immature state of processing, markers for the Golgi or trans-Golgi may be present or absent [27,40], (review [26]). When membranes are internalized by clathrin-mediated or clathrin-independent endocytosis and transported to the ERC, whatever contents arrived separately remain segregated in the ERC. Thus, the sorting function is done elsewhere [41]. These authors describe the ERC as a “complex combination of linked endosomal membranes and potentially independent structures” [42] (p. 109). As the perinuclear location is dictated by migration of vesicles on microtubules [43], the organelle is situated near the Golgi apparatus and might be a mixture of trans-Golgi vesicles with endosomes characterized by a slow mode of recycling. One of the problems in the field is that rates of vesicle trafficking are cell type-specific. Another problem is that the rate of recycling in all cells is typically faster, and the volume is greater, than generally acknowledged. The failure to recognize the lopsided kinetics of internalization and processing means that endocytosis is conflated with other processes in the literature, as mentioned elsewhere [15,43].

Despite the above argument, there are findings that support the concept that the ERC is a physiologically relevant compartment. Its vesicles are transported on microtubules, allowing its assembly around the centrosome. The resulting contraction of the ERC around the centrosome or its distribution out into cytoplasm [42] means it can interact with different subsets of compartments. Secondly, the ERC develops into a viral assembly compartment in cells infected with human cytomegalovirus [44], (review [45]). Not only is the ERC hijacked to assemble viral particles, but the new compartment then incorporates the cell’s machinery for autophagy [46]. A third argument is that PKC-dependent phosphorylation and glycosylation of EGFR at Thr654 dictate the route of EGF–EGFR complexes in the endosomal compartments [10,47]. That so many modifications near the juxtamembrane segment affect EGFR trafficking implies that small changes in trafficking, and therefore storage, could have a large impact on signaling. Indeed, there may be a biological function(s) that depends on the ERC, although the nature of the function is unknown.

We originally entertained the hypothesis that the ERC was a jumble of diverse vesicles that piled up near the centrosome due to bottlenecks in processing their contents and sending them on to lysosomes, trans-Golgi, or exocytotic vesicles. If the hypothesis is untenable, however, this raises the relationship between PKC and ERC to one with regulatory significance. Here, we find that endosomes trafficked to the interior are initially accompanied by active PKC. The results of Figure 3 suggest that this is processed into an inactive form that remains loosely associated with the vesicle. Although it is not known what molecule links PKC to the receptor complex, PKC-α [48] and PKC-δ [49] are known to be associated with actin on endosomes. Unlike PKC-ε, these isoforms do not contain any recognized actin-binding domain, and cortactin [50] and annexin [51] have been mentioned as possible means of binding PKC to the actin-rich coat. PKC-ε, as shown in Figure S2, is recruited to certain actin-rich scaffolds, either by this motif or in a similar way as PKC-α and PKC-δ, or both.

### 4.2. PKC Localization

We found that PKC was mainly cytoplasmic but was also localized to vesicles and actin-rich scaffolds, possibly including those on endocytic vesicles. The failure to identify these sites in other laboratories was due to the low-resolution imaging, which prevented previous workers from seeing small features. The cytoplasm also contains high levels of PKC. Two antibodies showed PKC-ε in different locations, raising the question of how these patterns of localization differed. It was known that the newly-synthesized form of PKC is bound on membranes (review [52]). This form is thought to be irrelevant in the current studies. In respiratory airway epithelial cells, the half-life of PKC is very long, and so the newly-synthesized molecules would constitute a small fraction of the total.

Although PKCs are activated at the plasma membrane (see below), the competent form, which is poised or “primed” for activation, is soluble. The competent form may exist as a homodimer that can be activated by DAG or phorbol esters [53] (Figure S1D). Phosphorylated sites important for PKC competency are exposed in the active enzyme; therefore, they can become dephosphorylated by a phosphatase [54]. This allows the PKC molecules to be recovered by a chaperone and rendered soluble again, as shown above (Figure 1B). In an alternate pathway [55], however, PKCs can be ubiquitinated and processed by an E3 ubiquitin ligase and finally degraded (Figure 1B). These PKC molecules would accompany the membrane as it was internalized into the cell, if we assume they are processed in the same way as other membrane-bound proteins [56]. Therefore, the PKC form that was found in the cytoplasm included enzymatically competent but inactive and possibly dimeric, molecules and inactive PKC molecules that were being recycled into the competent form. Although some vesicles were identified by both antibodies, most vesicles localized in the red channel were missing from the green and vice versa. This was thought to occur because the conformation that was tightly bound to newly-formed vesicles was rapidly processed into the inactive form.

Despite its recruitment to the membrane, PKC does not bind directly to EGFR. In extensive experiments on EGFR, recovering the receptor and its associated proteins by immunoprecipitation or immobilized metal affinity chromatography, the PKCs were rarely found. This was the case even when the phosphorylated peptides were recovered over a time course initiated by ligand binding or peptides with phosphoserine residues were recovered [57,58,59,60]. Nevertheless, the current results show that PKC attaches to sites where the EGFR resides and remains with the internalized EGFR. The conformation of PKC-ε that is tightly associated with minute vesicles is either enzymatically active or a form recently processed from the active form as shown in Figure 1B. Several laboratories have demonstrated the process of PKC translocation to the plasma membrane upon exposure to like activators or to DAG, using constructs of PKC with fluorescent proteins. In resting cells, PKC is mainly localized to the cytoplasm. Direct visualization of the process of PKC activation showed that different isoforms were translocated to the plasma membrane at times from 10 s to 15 min after stimulation [61,62,63,64]. In cells treated with thyrotropin releasing hormone, which activated signaling through endogenous molecules, a sequence of recruitment of different PKC isoforms was observed, and they remained active for varying durations of time [65].

It is assumed that, when colocalization of RR with the alternate form displaying the GG epitope was decreased, it was due to the fact that PMA treatment prolonged the association of active PKC with EGFR-bearing vesicles. A comparison of the antibodies themselves suggested they were superficially similar. According to the manufacturers, only one band was present on Western blots, and its molecular weight corresponded to PKC-ε. Secondly, a monoclonal antibody directed against amino acid residues 1–175 of PKC-ε was localized to both vesicles and actin filaments, as observed here with the polyclonal antibodies (data not shown). Thus, the antigen preference of the RR antibody is thought to reflect the opening up of the C-terminal upon activation. These newly available sites include the epitope in the sequence, 580–737. Both antigens were created in *Escherichia coli*, so the residue that was phosphorylated at the hydrophobic site, Ser729, in competent PKCs was not expected to be phosphorylated in either antigen (see Figure S1A,B).

The current results were interpreted in light of this fundamental information. The conformation of PKC displaying the RR (R&D, red) epitope was tightly bound to vesicles underlying the plasma membrane. Thus, RR must be the active form or at least a membrane-bound molecule undergoing processing by E3 ligase. Whereas it was not possible to discern which of the PKC conformations was localized by the GG antibody, they were all inactive. Thus, we could compare active and inactive PKC in control cells as well as cells treated with the PKC activator, PMA.

There was previous evidence that vesicles retained activated PKC and then assembled actin comets which could propel the vesicles. The same investigators discovered that this mechanism could be reconstituted in vitro by adding Cdc42 and N-WASP [51]. Not only endosomes, but also multivesicular bodies and lysosomes, recruited PKC and assembled actin. It has been noted that early endosomes and recycling endosomes have phosphatidylserine. When combined with DAG, the phosphatidylserine binding site of PKC would help retain the protein on these vesicles. These adhesive sites would be lost as vesicles matured during trafficking, and the phosphatidylserine content was lowered. This, along with the processing of the PKC molecule, illustrated in Figure 1B, explains the rise in the GG form of PKC in vesicles farther from the cell edge. On the contrary, PMA treatment maintains the activity and decreases the processing. PKC has over 800 documented substrates [66], so it is possible that critical substrates are phosphorylated and continue on the endosomes directing them to perinuclear vesicles. In other words, PKC may alter trafficking through the conservation of enzymatic activity, and perhaps also through an ability to initiate actin scaffolds.

### 4.3. Relevance to Growth Control and Cancer

The relevance of EGFR trafficking extends beyond the initial trafficking and signaling output of the receptor, because EGFR is well-known to be activated by other receptors. One example is the GPCR, GPR30 (GPER, G protein-coupled estrogen receptor), which transactivates EGFR by causing release of heparan-bound EGF [67]. The GPCRs also initiate DAG production through PLCβ activation, as shown in Figure 1A. Thus, a GPCR that initiates transactivation is likely to stimulate PKC activation early and only later advance the transactivation and PKC production that normally results from EGFR signaling. Early PKC activation may cause rapid phosphorylation of the EGFR and thereby cause desensitization. On the other hand, activation sustained by signaling downstream of the EGFR may cause the redirection of vesicle trafficking in a similar way as shown in the current work. The cross-talk among signaling pathways very likely hinges on the temporality of PKC interaction with the entire population of receptors. Thus, better methods of predicting the outcomes of this cross-talk are required before the impact on cell growth control can be understood.

## 5. Conclusions

The relationship between receptor-mediated signaling and regulation of vesicle trafficking is an important issue in cancer therapy. Antibodies against members of the EGFR family are used in cancer therapy, and it has been shown that they cause internalization of the receptors. Initially, the strategy was to prevent excessive growth factor signaling and tumor growth. It seems that internalization by itself does not block signaling, which may therefore persist in some internal compartments. It is paramount to gain an understanding of receptor traffic patterns along with their regulatory mechanisms. PKC phosphorylates EGFR and then accompanies the vesicles bearing EGF-tagged gold particles into the ERC. The trafficking patterns, however, depend on the processing of enzymatically active PKC into inactive PKC. If cells are treated with a PKC activator, the vesicles are less likely to display the inactive form of PKC and more likely to enter the ERC.

## Supplementary Materials

The following are available online at https://www.mdpi.com/2218-273X/10/9/1288/s1, Figure S1: Characterization of anti-PKC-ε antibodies. Figure S2: PKC colocalizes with vinculin in loosely textured, actin-based features. Figure S3: Negative controls on 1000 W cells. Video S1: Stack 3F video for comparison to Figure 3F.
