# Supplementary material for: Activated Protein Kinase C (PKC) Is Persistently Trafficked with Epidermal Growth Factor (EGF) Receptor
Source: Biomolecules. 2020 Sep 7;10(9):1288. doi: 10.3390/biom10091288 (PMC7563713; doi:10.3390/biom10091288)
Supplement: Supplementary file 1 [file biomolecules-10-01288-s001.pdf]

SUPPLEMENTAL MATERIAL (Heckman, Biswas, Dimick, and Cayer, 2020)

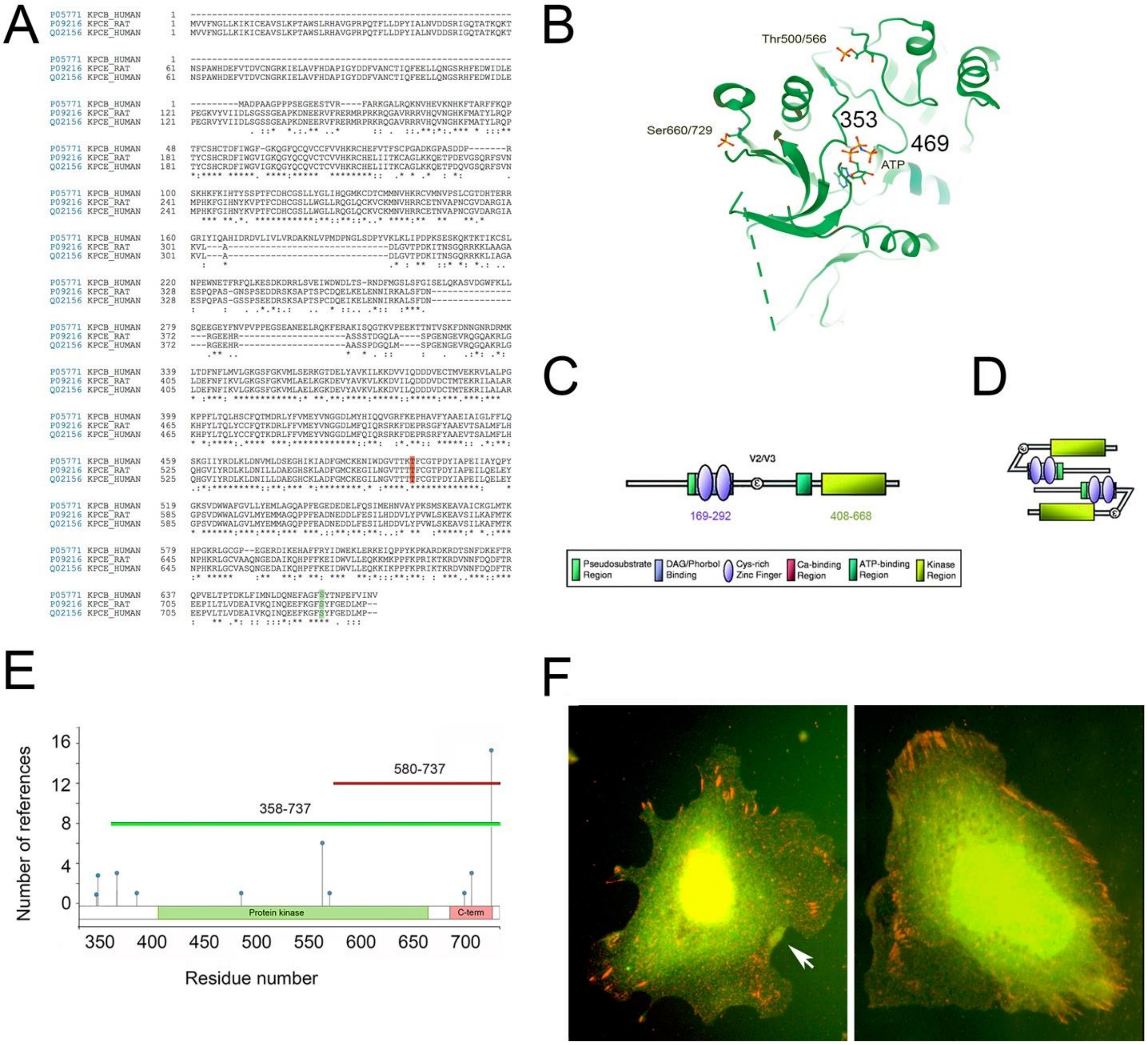

Figure S1. Characterization of anti-PKC–ε antibodies. The antibodies were directed against recombinant fragments that contained the residues usually phosphorylated (Thr566, corresponding to Thr500 in PKC-βII, and Ser729, corresponding to S660 in PKC–β). (A) Alignment of PKC–β and PKC–ε sequences with colored columns indicating the Thr500 and Ser660 residues, (B) Cut-away view of the PKC–βII active site, showing Thr500 and Ser660 phosphorylated residues positioned near the ATP binding site, (C, D) Domain organization of PKC-ε with a possible homodimer similar to that formed by PKC–α [33], (E) Map showing the long and short sequences of unphosphorylated PKC–ε residues housing the GG and RR epitopes respectively. (F) Comparison of cells before and after 10 h of exposure to phorbol ester. In the cell on left (0 h), PKC–ε is localized to a cloud-like feature (arrow) and minute granules. The cell on right, treated for 10 h, shows mainly diffuse PKC–ε. Cells were stained with anti-PKC GG (green) and anti-vinculin (red). PKC–βII active site model was from PDB3PFQ [34]. The graphic of C was modified from [35].

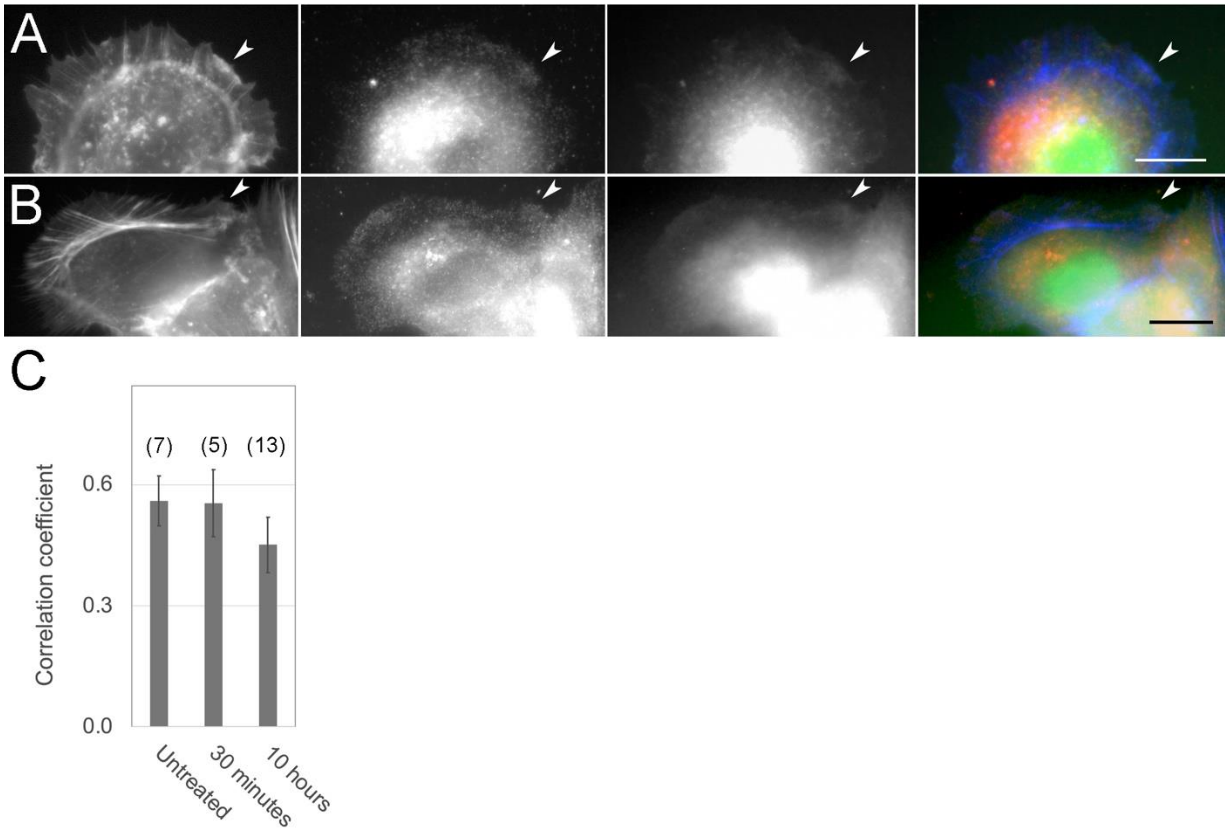

Figure S2. PKC colocalizes with vinculin in loosely textured, actin-based features. (A-B) Actin (left panel, blue), vinculin (middle, red), PKC (right, green), and color overlay showing all three proteins (arrowheads). (A) All three proteins are present on a ruffle. (B) All three proteins are present in features at the cell edge that are cloud-like but do not seem to be ruffles. (C) Correlation coefficients between PKC and vinculin in thin, peripheral areas from images. The samples are untreated or exposed to phorbol ester activator of PKC for the indicated times. This confirms that their relationship is unaltered by PMA treatment. The number of images sampled is given in parentheses. Bar = 10 μm.

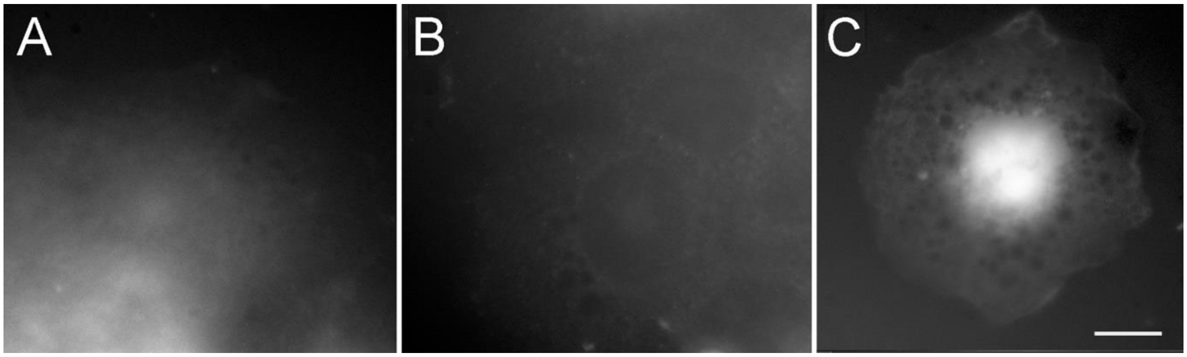

Figure S3. Negative controls on 1000 W cells. (A-B) Controls on the procedure of Figure 1. (A) Secondary goat fluorescein isothiocyanate-labeled anti-rabbit staining following an irrelevant primary antibody. (B) Secondary donkey anti-mouse TRITC-labeled antibody staining following an irrelevant primary antibody. (C) NanoPartz gold particles conjugated with Cy3-tagged goat anti-mouse immunoglobulin G, added to cells for 40 minutes at 4°C, and then rewarmed in Wich+10% medium for 5 min. This is a control on the procedure used for Figure 2. Bar = 10 μm.
